# Chronic use of inhaled corticosteroids in patients admitted for respiratory virus infections: a 6-year prospective multicenter study

**DOI:** 10.1038/s41598-022-08089-0

**Published:** 2022-03-10

**Authors:** David Luque-Paz, Pierre Tattevin, Paul Loubet, François Bénézit, Vincent Thibault, Fabrice Lainé, Philippe Vanhems, Selilah Amour, Bruno Lina, Xavier Duval, Anne-Sophie L’Honneur, Nadhira Fidouh, Christine Vallejo, Sophie Alain, Florence Galtier, Vincent Foulongne, Gisèle Lagathu, Nezha Lenzi, Zineb Lesieur, Odile Launay, Stéphane Jouneau, the FLUVAC Study Group, O. Launay, N. Lenzi, Z. Lesieur, P. Loulergue, S. Momcilovic, J. P. Mira, N. Marin, J. Charpentier, A. Regent, R. Kanaan, F. Dumas, B. Doumenc, A. S. L’Honneur, M. Lachatre, T. Szwebel, J. Kansao, Y. Costa, X. Duval, J. F. Alexandra, H. Becheur, K. Belghalem, J. Bernard, A. Bleibtreu, M. Boisseau, R. Bories, O. Brugiere, F. Brunet, C. Burdet, E. Casalino, M. Caseris, C. Chansiaux, M. Chauchard, P. Chavance, C. Choquet, A. Cloppet-Fontaine, L. Colosi, B. Couset, B. Crestani, F. Crocket, A. Debit, Delanoe, V. Descamps, P. Dieude, A. Dossier, N. Douron, E. Dupeyrat, N. Emeyrat, C. Fernet, T. Goulenok, S. Harent, R. Jouenne, A. Justet, M. Lachatre, A. Leleu, I. Lerat, M. Lilamand, H. Mal, A. Marceau, A.-C. Metivier, K. Oplelatora, T. Papo, A.-L. Pelletier, L. Pereira, P. Pradere, Prommier, P. Ralainnazava, M. Ranaivoision, A. Raynaud-Simon, C. Rioux, K. Sacre, V. Verry, V. Vuong, Y. Yazdapanah, N. Houhou, F. Galtier, P. Géraud, V. Driss, V. Maugueret, L. Crantelle, C. Agostini, M. Ray, F. Letois, T. Mura, C. Serrand, C. Agostini, S. Noslier, A. Giordano, H. Chevassus, E. Nyiramigisha, C. Merle, A. Bourdin, A. Konaté, X. Capdevilla, G. Du Cailar, A. Terminet, H. Blain, M. S. Leglise, A. Le Quellec, P. Corne, L. Landreau, K. Klouche, A. Bourgeois, M. Sebbane, G. Mourad, H. Leray, V. Foulongne, D. Postil, S. Alcolea, E. Couve-Deacon, S. Rogez, S. Amour, P. Vanhems, L. Argaud, M. Cour, R. Hernu, M. Simon, T. Baudry, K. Tazarourte, C. Bui-Xuan, J. Fattoum, B. Lina, M. Valette, F. Lainé, V. Thibault, S. Rochas, S. Cochennec, E. Thébault, G. Lagathu, S. Jouneau, M. Revest, F. Bénézit, M. Sébillotte, A. Le Bot, M. Baldeyrou, S. Patrat-Delon, M. Cailleaux, C. Pronier, P. Tattevin

**Affiliations:** 1 Service de Pneumologie, Hôpital Pontchaillou, CHU Rennes, Rennes, France; 2 Service de Maladies Infectieuses et Réanimation Médicale, Hôpital Pontchaillou, CHU Rennes, Rennes, France; 3 Inserm U1230, Université Rennes‑I, Rennes, France; 4 Inserm CIC 1414, CHU Rennes, Rennes, France; 5 Department of Infectious and Tropical Diseases, CHU Nîmes, Univ Montpellier, Nîmes, France; 6 Inserm U1047, University of Montpellier, Nîmes, France; 7 Inserm, F-CRIN, Réseau Innovative Clinical Research in Vaccinology (I-REIVAC), Paris, France; 8 Virologie, Hôpital Pontchaillou, CHU Rennes, Rennes, France; 9 Service Hygiène hospitalière, Epidémiologie et Prévention, Hospices Civils de Lyon, Lyon, France; 10 Laboratoire des Pathogènes Emergents-Fondation Mérieux, Centre International de Recherche en Infectiologie, Centre National de la Recherche Scientifique, Institut National de la Santé et de La Recherche Médicale U1111, UMR5308, Ecole Normale Supérieure de Lyon, Université Claude Bernard Lyon, Lyon, France; 11 Laboratoire de Virologie, Institut des Agents Infectieux (IAI), Centre National de Référence des Virus Respiratoires France Sud, Hôpital de la Croix-Rousse, Hospices Civils de Lyon, Lyon, France; 12 CIC1125, Hôpital Bichat Claude Bernard, Paris, France; 13 Service de Virologie, Hôpital Cochin, Paris, France; 14 Laboratoire de Virologie, Hôpital Bichat, AP-HP, Paris, France; 15 Inserm CIC 1435, CHU Dupuytren, Limoges, France; 16 Inserm U1092, CHU Limoges, RESINFIT, Limoges, France; 17 Inserm CIC 1411, Hôpital Saint Eloi, CHU Montpellier, Montpellier, France; 18 Service de Virologie, Hôpital Saint Eloi, CHU Montpellier, Montpellier, France; 19 Faculté de Médecine Paris Descartes, Université de Paris, Paris, France; 20 Hôpital Cochin, Assistance Publique Hôpitaux de Paris, Paris, France; 21 Inserm CIC 1417, Paris, France; 22 Inserm UMR1085 IRSET, Université de Rennes 1, EHESP, Rennes, France; 23 Hôpital Cochin, Paris, France; 24 Hôpital Bichat Claude-Bernard, Paris, France; 25 CHU de Montpellier, Montpellier, France; 26 CHU Dupuytren, Limoges, France; 27 Hospices Civils de Lyon, Lyon, France; 28 CHU Pontchaillou, Rennes, France

**Keywords:** Infectious-disease diagnostics, Virology, Risk factors

## Abstract

Inhaled corticosteroids (ICS) have been associated with increased risk of pneumonia. Their impact on respiratory virus infections is unclear. We performed a post-hoc analysis of the FLUVAC cohort, a multicenter prospective cohort study of adults hospitalized with influenza-like illness (ILI) during six consecutive influenza seasons (2012–2018). All patients were tested for respiratory virus infection by multiplex PCR on nasopharyngeal swabs and/or bronchoalveolar lavage. Risk factors were identified by logistic regression analysis. Among the 2658 patients included, 537 (20.2%) were treated with ICS before admission, of whom 282 (52.5%, 282/537) tested positive for at least one respiratory virus. Patients on ICS were more likely to test positive for non-influenza respiratory viruses (25.1% vs. 19.5%, *P* = 0.004), especially for adenovirus (aOR 2.36, 95% CI 1.18–4.58), and respiratory syncytial virus (aOR 2.08, 95% CI 1.39–3.09). Complications were reported in 55.9% of patients on ICS (300/537), primarily pneumonia (171/535, 32%). Among patients on chronic ICS who tested positive for respiratory virus, 14.2% (40/282) were admitted to intensive care unit, and in-hospital mortality rate was 2.8% (8/282). Chronic use of ICS is associated with an increased risk of adenovirus or RSV infections in patients admitted for ILI.

## Introduction

Inhaled corticosteroids (ICS) are commonly used for the treatment of various chronic respiratory diseases, including asthma, and chronic obstructive pulmonary diseases (COPD)^1^. In 2018, the number of patients with COPD or asthma was estimated at 600 million worldwide^2–4^. A recent study showed an association between chronic respiratory diseases, and the risk of non-influenza respiratory virus (NIRV) infections, but did not address ICS use^5^. Several randomized studies demonstrated that appropriate use of ICS improves respiratory function and quality of life, and reduces the risk of exacerbations^6^. However, chronic use of ICS has been associated with an increased risk of pneumonia^7–11^. Few studies evaluated the risk of viral infections in patients with chronic use of ICS. A landmark study demonstrated that ICS inhibit antiviral activity in situ, leading to delayed virus clearance and increased bacterial load during COPD exacerbations^12^. However, clinical data on the potential association between chronic use of ICS, and the risk of respiratory virus infections, are scarce. We aimed to compare the characteristics and outcome of respiratory virus infections in adults hospitalized for influenza-like illness (ILI) with, or without, chronic use of ICS.

## Materials and methods

### Study design

We performed a post-hoc analysis of the FLUVAC study, a multicenter prospective cohort of adult patients admitted for influenza-like illness in six French university hospitals^13^: Cochin Hospital, Paris; Bichat Hospital, Paris; Pontchaillou Hospital, Rennes; Dupuytren Hospital, Limoges; Montpellier University Hospital; Edouard Herriot Hospital, Lyon. During the study period, all adults hospitalized for at least 24 h with ILI during the influenza season in France (December-March), with symptoms onset < 7 days before screening, were invited to participate. ILI was defined as a combination of two criteria: (i) at least one of the following symptoms: fever (≥ 38 °C), headache, myalgia or malaise, and (ii) at least one of the following respiratory symptoms: cough, sore throat, or shortness of breath (dyspnea). The characteristics and outcome of patients with influenza, respiratory syncytial virus (RSV), and other NIRV in this cohort have been previously reported^5,13–15^. Data on demographic characteristics, comorbidities, treatment before admission (including ICS), clinical presentation, testing for respiratory viruses, hospitalization, treatment, and outcome, were prospectively collected on a standardized questionnaire, from medical charts, and through face-to-face interviews with patients.

For the study reported herein, we included all patients enrolled during the first six FLUVAC seasons (2012/13, 2013/14, 2014/15, 2015/16, 2016/17, and 2017/18). Patients with missing data about ICS use, or testing for respiratory viruses, were excluded.

### Virological data

All patients were tested for a panel of respiratory viruses, mostly by nasopharyngeal swabs, and in some cases by bronchoalveolar lavage, if clinically indicated. Respiratory samples were initially tested in the virology units of the participating hospitals by in-house real-time influenza A & B PCR after manual nucleic acid extraction. Amplification was performed with ABI 7500 thermocycler. All samples were then sent to the French National Reference Center for respiratory viruses (CNR-Lyon) for confirmation by RT-PCR. Samples were also screened for a panel of NIRV: adenovirus (52 serotypes), human bocaviruses 1–4, human coronaviruses 229E, NL63, OC43, and HKU1, human metapneumoviruses A1, A2, B1 and B2, parainfluenza viruses 1–4, picornavirus, and respiratory syncytial virus (RSV), by real-time PCR, using the Respiratory Multiwell System (MWS) r-gene assay (bioMérieux S.A., Marcy l’Etoile, France) on an ABI 7300 analyzer.

### Ethics

The FLUVAC study (clinicaltrials.gov NCT02027233) was performed in accordance with the principles of Good Epidemiological and Clinical Practices in clinical research and the Declaration of Helsinki, and the study protocol was approved by regional ethics committees (Comité de Protection des Personnes ‘Ile-de-France IV’). This ancillary study was approved by the institutional review board of I-REIVAC (Innovative Clinical Research Network in Vaccinology, France). All the study participants provided written informed consent for respiratory virus testing and data collection before inclusion.

### Statistical analysis

We performed a descriptive analysis of the total population and of the population of patients positive for at least one respiratory virus, according to chronic use of ICS. Results were expressed as mean and standard deviation (SD), or median and interquartile range (IQR) for quantitative variables, and n (%) for qualitative variables. The Student test or Fisher’s exact test was used, as appropriate, for univariate comparisons. Missing data for each variable were excluded from the denominator.

To evaluate a potential association between ICS and respiratory virus infections, we performed a multivariate analysis, using a backward logistic regression model for each virus associated with ICS in univariate analysis. In each virus model, associated factors with a *P*-value < 0.2 in univariate analysis were included in the multivariate analysis. The multivariate analysis for each virus was also adjusted for age and sex. We performed a Bonferroni correction in order to maintain a global alpha risk at 5%. A *P*-value of 0.05 or less was considered statistically significant. Variables with > 10% missing values were excluded from multivariate analyses with one exception for ‘chronic pulmonary disease’ (27% missing values) which is known to be a major risk factor for viral infections. Collinearity was assessed using the variance inflation factor (VIF) < 5.

Finally, we performed multivariate analyses using a backward stepwise logistic regression model using intensive care unit (ICU) admission as the dependent variable and including covariates with a *P-*value < 0.2 in univariate analysis, namely age (continuous variable), influenza vaccine, systemic corticosteroids, ICS, empirical antiviral treatment, RSV infection, and influenza virus infection. The model was adjusted for other covariates known to influence ICU admission: chronic heart disease, chronic heart failure, immunosuppressive treatment and malignancy.

Results from both regressions were expressed as odds ratios (OR) and adjusted odds ratios (aOR) with their 95% confidence intervals (95% CI). All analyses were performed using R-Studio (Integrated Development for R. RStudio, Inc., Boston, USA).

## Results

### Patients characteristics and virus distribution

**Figure 1:**
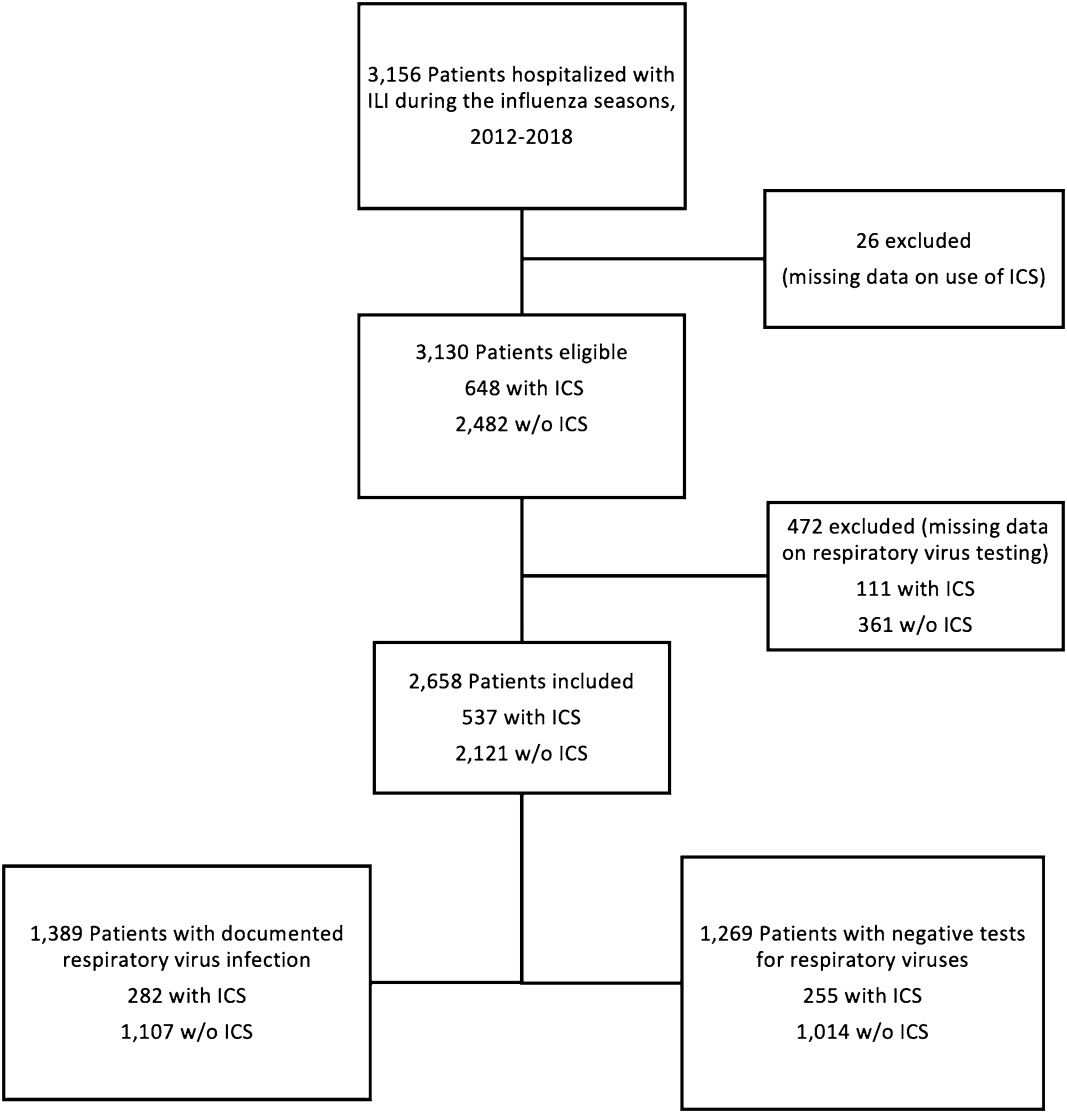
Study flow chart. *ILI* influenza-like illness, *ICS* inhaled corticosteroids.

Of the 3156 patients included in the FLUVAC study, 2658 (84.2%, 95% CI 49–53) had complete data on chronic use of ICS, and results of respiratory viruses testing (Fig. 1). The median age was 71 years [IQR 56–83], 53.7% were men, 81.6% had at least one chronic underlying disease, 45.7% had been hospitalized in the previous 12 months, and 46.9% had been vaccinated against influenza during the last 6 months. At least one respiratory virus was found in 52.2%, mostly influenza (64.4% of all respiratory viruses, 895/1389), picornavirus (11.2%, 156/1389), RSV (9.2%, 128/1389), coronavirus (8.6%, 120/1389), human metapneumovirus (6.8%, 94/1389), adenovirus (2.7%, 38/1389), bocavirus (1.7%, 23/1389), and parainfluenza virus (1.2%, 16/1389). At least 2 respiratory viruses were identified in 84 patients (6%).

### Characteristics of patients with chronic use of inhaled corticosteroids

Of the 2,658 patients enrolled, 537 patients (20.2%) were chronic users of ICS (Supplementary Table S1). These patients had a median age of 70 years [IQR 58–81], and 283 (52.7%) were men. The main comorbidities in patients on chronic use of ICS was chronic respiratory disease (89.3%), chronic heart disease (42.2%), diabetes (22%), haematological or solid malignancy (16.3%), and chronic kidney disease (13.2%). Of note, 19.4% of patients on chronic use of ICS were also taking systemic corticosteroids. However, in all analyses performed, there was no collinearity between ICS and systemic corticosteroids. Main symptoms were dyspnea (89.6%), fever (84.5%), cough (76%), and myalgia (22%). Median duration of ILI symptoms before admission was 2 days [1–3]. The median length of hospital stay was 7 days [IQR 4–7]. A total of 300 patients presented at least one complication (55.9%) during their hospital stay, including respiratory failure (37.8%), pneumonia (32%), mechanical ventilation (14.8%), heart failure (14%), renal failure (11.8%), acute respiratory distress syndrome (9%), and shock (1.7%). ICU admission occurred in 67 patients (12.5%). In-hospital mortality was 3.2%.

As compared to patients not on ICS, patients with chronic use of ICS were more likely to receive systemic corticosteroids (19.4% vs. 9.6%, *P* < 0.001), and to have received an influenza vaccine during the last 6 months (59.6% vs. 43.7%, P < 0.001). Dyspnea was more frequent on admission in patients with chronic use of ICS (86.7% vs. 73.1%, *P* < 0.001).

In univariate analysis, chronic use of ICS was associated with higher risk of respiratory failure (37.8% vs. 27.2%, *P* < 0.001), and ICU admission (12.5% vs. 8.4%, *P* = 0.01). After adjustment for age, sex, comorbidities, empirical antiviral treatment and respiratory virus, these associations between ICS use and ICU admission or respiratory failure were no longer present (Supplementary Table S2). In-hospital mortality was 3.2% (17/537) in patients with chronic use of ICS, and 4.5% (96/2121) in patients with no use of ICS (*P* = 0.18).

### Patients with respiratory virus infections: comparison of patients with or without chronic use of inhaled corticosteroids (Tables 1, 2)

**Table 1:** Patients with documented respiratory virus infections: comparison of those with, or without chronic use of inhaled corticosteroids (ICS).

	Chronic use of ICS N = 282	No use of ICS N = 1107	*P*-value
**Number included each season**			
2012/13	42 (14.9)	163 (14.7)	
2013/14	44 (15.6)	132 (12)	
2014/15	32 (11.3)	198 (17.8)	
2015/16	45 (15.9)	167 (15.1)	
2016/17	57 (20.3)	200 (18.1)	
2017/18	62 (22)	247 (22.3)	
**Baseline characteristics**			
Median age [IQR]	69 [56–80.8]	72 [56–83]	0.16
Age > 65 years, n (%)	170/282 (60.3)	695/1107 (62.8)	0.45
Men, n (%)	146/282 (51.8)	578/1107 (52.2)	0.90
Median BMI, kg/m^2^ [IQR]	24.8 [21.3–28.1]	24.9 [21.6–28.4]	0.79
Chronic diseases			
Chronic respiratory disease, n (%)	175/196 (89.3)	251/812 (30.9)	< 0.001
Chronic heart disease, n (%)	120/280 (42.9)	448/1106 (40.5)	0.50
Chronic kidney disease, n (%)	38/281 (13.5)	150/1106 (13.6)	1
Splenic dysfunction, n (%)	17/280 (6.1)	49/1105 (4.4)	0.27
Cirrhosis, n (%)	8/281 (2.8)	34/1106 (3.1)	1
Malignancy, n (%)	42/281 (14.9)	189/1105 (17.1)	0.42
Mellitus diabetes, n (%)	60/282 (21.3)	237/1106 (21.4)	1
Smoking status, n (%)			
Active smoking	49/282 (17.4)	159/929 (17.1)	0.22
Ex-smoker (> 1 year)	103/242 (42.5)	285/929 (30.7)	
No smoker	90/242 (37.1)	485/929 (52.2)	
Current influenza vaccination, n (%)	153/282 (54.3)	448/1097 (40.8)	< 0.001
Associated treatment			
Systemic corticosteroids, n (%)	53/282 (18.8)	124/1107 (11.2)	0.001
Immunosuppressive drugs, n (%)	24/281 (8.5)	112/1106 (10.1)	0.50
**Clinical presentation**			
Median time from symptom onset to hospitalization, days [IQR]	2 [1–3]	2 [1–3]	0.56
Fever, n (%)	247/282 (87.6)	965/1105 (87.3)	1
Myalgia, n (%)	79/281 (28.1)	283/1095 (25.8)	0.44
Cough n (%)	228/282 (80.9)	947/1105 (85.7)	0.051
Dyspnea, n (%)	170/196 (86.7)	591/808 (73.1)	< 0.001
**Outcome and treatment**			
In-hospital all causes of mortality, n (%)	8/282 (2.8)	53/1107 (4.8)	0.24
ICU admission, n (%)	40/282 (14.2)	89/1107 (8.0)	0.002
Median length of stay, days [IQR]	7 [4–12]	6 [3–10]	0.08
Complication			
Pneumonia, n (%)	89/281 (31.7)	332/1103 (30.1)	0.62
Respiratory failure, n (%)	102/281 (36.3)	314/1103 (28.5)	0.01
ARDS, n (%)	24/281 (8.5)	105/1102 (9.5)	0.73
Heart failure, n (%)	41/281 (14.6)	150/1101(13.6)	0.70
Renal failure, n (%)	34/281 (12.1)	153/1103 (13.9)	0.49
Shock state, n (%)	8/281 (2.8)	45/1102 (4.1)	0.39

Data are given as n (%) or median [interquartile range].

*IQR* interquartile range, *BMI* body mass index, *ARDS* acute respiratory distress syndrome.

**Table 2:** Respiratory virus infections in patients with, or without chronic use of inhaled corticosteroids (ICS).

Viral documentation	Chronic use of inhaled corticosteroids (ICS), N = 282	No use of ICS N = 1107	OR (95% CI)	*P*-value	Adjusted OR^1^ (95% CI)	*P*-value^2^
	n/N (%)	n/N (%)				
**Influenza**	159/282 (56.4)	736/1107 (66.5)	0.65 (0.50–0.85)	0.002	0.86 (0.6–1.24)^a^	1
Influenza A	117/159 (73.6)	531/736 (72.2)				
Influenza B	43/159 (27.0)	206/736 (28)				
Adenovirus	14/282 (5.0)	24/1107 (2.2)	2.36 (1.17–4.56)	0.013	2.36 (1.18–4.58)^b^	0.036
Bocavirus	3/274 (1.1)	20/1086(1.8)	0.59 (0.14–1.74)	0.397		
Coronavirus	33/179 (18.4)	87/649 (13.4)	1.46 (0.93–2.25)	0.092		
Metapneumovirus	17/282 (6.0)	77/1103 (7.0)	0.85 (0.48–1.43)	0.57		
Parainfluenza virus	6/197 (3.0)	10/811 (1.2)	2.52 (0.85–6.86)	0.078		
Picornavirus	32/279 (11.5)	124/1098 (11.3)	1.02 (0.66–1.52)	0.93		
RSV	41/282 (14.5)	87/1104 (7.9)	1.99 (1.33–2.94)	0.0007	2.08 (1.39–3.09)^c^	0.001
Coinfection	22/282 (7.8)	62/1107 (5.6)	1.43 (0.82–2.40)	0.16		

Logistic regression analysis.

*OR* odds-ratio, *95% CI* 95% confidence interval.

^1^Multivariate analysis included all variables with P < 0.2 for each virus as well as sex and age (continuous variable).

^2^After Bonferroni correction.

^a^For influenza, stepwise backward analysis included ICS, influenza vaccine during the last 6 months, diabetes, malignancy, chronic pulmonary disease, as well as sex and age. The final model included chronic pulmonary disease, malignancy, seasonal influenza vaccination, age and sex.

^b^For adenovirus, stepwise backward analysis included ICS, diabetes, chronic pulmonary disease as well as sex and age. The final model included ICS, age and sex.

^c^For RSV, stepwise backward analysis included ICS, malignancy, chronic pulmonary disease, chronic heart disease, chronic kidney disease, systemic corticosteroids as well as sex and age. The final model included ICS, malignancy, sex and age.

The proportion of documented respiratory virus infections was similar between patients with, or without, ICS (282/537, 52.5% vs. 1107/2121, 52.2%; *P* = 0.92). However, patients on ICS were more likely to test positive for NIRV (135/537, 25.1% *vs*. 413/2121, 19.5%, *P* = 0.004), and less likely to test positive for influenza virus (159/282, 56.4% vs. 736/1107, 66.5%, *P* = 0.002). Proportion of A/B influenza viruses was similar in patients with, or without ICS. Patients on ICS were more likely to test positive for adenovirus (14/282, 5% *vs*. 24/1107, 2.2%, *P* = 0.013), and RSV (41/282, 14.5% vs. 87/1104, 7.9%, *P* = 0.001). At least 2 viruses were documented in 22 patients (7.8%) in the ICS group, and 62 patients (5.6%) in the group without ICS. On multivariate analysis, ICS use was significantly associated with adenovirus (aOR 2.36, 95% CI 1.18–4.58), and RSV (aOR 2.08, 95%CI 1.39–3.09), but not with influenza (aOR 0.86, 95% CI 0.6–1.24) (Supplementary Table S3).

## Discussion

In this post-hoc analysis of 2,658 adult patients hospitalized for community-acquired ILI and tested for respiratory virus infections by multiplex PCR, we found that chronic use of ICS was associated with increased risk of NIRV infections, particularly adenovirus and RSV. Although patients on ICS were more likely to be transferred in ICU, in-hospital mortality rates were similar in patients with, or without, ICS, and chronic use of ICS was not associated with ICU admission on multivariate analysis.

Only few studies have evaluated the impact of chronic use of ICS on the characteristics and outcomes of respiratory virus infections. ICS have been associated with higher bacterial load in sputum^16^, and increased risk of pneumonia^10,11,17,18^. In the latter studies, the diagnosis of pneumonia was based on radio-clinical criteria, with no or limited microbiological documentation. Two recent meta-analyses^7,8^, and one narrative review^9^, confirmed that chronic use of ICS is associated with increased risk of pneumonia overall, but could not specify whether this applies for virus as well as for bacteria. Of note, chronic use of ICS has been convincingly associated with a higher risk of non-tuberculous mycobacterial infections^19–21^.

Singanayagam et al*.* showed that ICS impair innate and acquired antiviral immune responses, through an alteration of interferon production, and antimicrobial peptides deficiency, leading to delayed virus clearance^12^. At least one respiratory virus has been documented in up to 45% of COPD exacerbations in patients treated by ICS, primarily rhinovirus, adenovirus, and influenza^16^. A retrospective multicenter study reported 15 consecutives cases of severe pneumonia due to adenovirus in immunocompetent patients, of whom 14 were on ICS^22^. Of note, human adenoviruses are commonly associated with severe respiratory infections, even lethal, in immunocompromised but also in immunocompetent patients^23,24^.

To the best of our knowledge, no clinical study found an increased risk of RSV in patients on chronic use of ICS. However, this association is biologically plausible, as human cathelicidin LL-37, which inhibits RSV in vitro and in vivo^25^, is suppressed by ICS^26^. The increased risk of RSV infections in patients with malignancy, immunosuppression and/or chronic pulmonary disease, as pointed out by our multivariate analysis, has been documented by others^14,27,28^. Although influenza was more rarely identified in patients treated by ICS (56.4% vs. 66.5%, *P* = 0.002), this association was no longer significant in multivariate analysis, and could be explained by the higher influenza vaccination coverage in patients on ICS. Indeed, yearly vaccination against seasonal influenza is recommended in most patients with chronic use of ICS, as these treatments are primarily prescribed in patients with chronic pulmonary diseases.

We found no differences in the occurrence of coronaviruses infections between patients treated by ICS and those who had not. Our data were from before the COVID-19 pandemic and we only studied human coronaviruses 229E, NL63, OC43, and HKU1. These results could be different with SARS-CoV-2 because it was recently demonstrated that ICS downregulated the bronchial epithelial expression SARS-CoV-2 related genes, especially *ACE2* that encodes the SARS-CoV-2 human receptor^29^. To date, studies that have evaluated the impact of ICS on COVID-19 clinical outcomes found discrepant results^30–32^.

This study has limitations. First, given that the analysis of ICS impact was not the primary objective of the FLUVAC prospective cohort study, treatment by ICS was only collected as a dichotomic variable (yes/no), with no information on dose, duration, and comedication. Hence, we were unable to analyze a potential dose–response effect, as documented for the association between ICS and pneumonia^11^. Likewise, we could not compare the impact of different ICS, while fluticasone has been associated with higher risk of pneumonia than budesonide^8,11^. Second, data on underlying respiratory diseases have not been collected as well, so that we could not compare respiratory virus distribution, and its association with ICS, in patients with COPD, or asthma, the main indications for ICS. Third, the study design did not allow us to measure prevalence or incidence rate in population treated by ICS. Fourth, our study was performed in a single country in Western Europe, so that our findings may not apply to other countries with different epidemiology of respiratory viruses, or other practices regarding ICS use and influenza vaccination policies. Also, there are discrepancies between the ECDC, WHO and CDC definitions of ILI, which could restrict the generalization of our results. Of note, our findings could be seasonally biased, especially for picornaviruses (enteroviruses and rhinovirus) which may circulate anytime during the year, while our study was restricted to influenza seasons. Fifth, we have no robust data to support causality between respiratory virus detection and ILI in the patients enrolled. However, our study has strengths, including its prospective, multicenter design and standardization of viral test for all patients included, during six consecutive years.

In conclusion, this study suggests a potential link between chronic use of ICS, and respiratory virus infections. To our knowledge, no previous study identified ICS as a risk factor for adenovirus and RSV infections. Further studies are needed to evaluate the role of ICS on viral immunity and their impact on respiratory infections.

## Supplementary Information

Supplementary Tables.

## Abbreviations

ARDS: Acute respiratory distress syndrome
BMI: Body mass index
COPD: Chronic obstructive pulmonary disease
ICS: Inhaled corticosteroids
ICU: Intensive care unit
ILI: Influenza-like illness
IQR: Interquartile range
NIRV: Non-influenza respiratory viruses
RSV: Respiratory syncytial virus
SD: Standard deviation
