# Supplementary material for: Chronic use of inhaled corticosteroids in patients admitted for respiratory virus infections: a 6-year prospective multicenter study
Source: Sci Rep. 2022 Mar 10;12:4199. doi: 10.1038/s41598-022-08089-0 (PMC8913614; doi:10.1038/s41598-022-08089-0)
Supplement: Supplementary file 1 — Supplementary Tables. [file 41598_2022_8089_MOESM1_ESM.docx]

**Supplementary Table S1.** Characteristics and outcomes of all patients admitted for influenza-like illness

| Number included each season | Chronic use of ICS,  n=537 | No use of ICS,  n=2121 | Patients  excluded, n=472 |
| --- | --- | --- | --- |
| 2012/13 | 77 (14.3) | 333 (15.7) | 33 (7.0) |
| 2013/14 | 95 (17.7) | 289 (13.6) | 20 (4.2) |
| 2014/15 | 53 (9.9) | 311 (14.7) | 233 (49.4) |
| 2015/16 | 89 (16.6) | 366 (17.3) | 3 (0.6) |
| 2016/17 | 98 (18.2) | 327 (15.4) | 86 (18.2) |
| 2017/18 | 125 (23.3) | 495 (23.3) | 88 (18.6) |
| **Baseline characteristics** |  |  |  |
| Median age, years [IQR] | 70 [58-81] | 71 [56-83] | 73 [59-82] |
| Men, n (%) | 283/537 (52.7) | 1144/2121 (53.9) | 263/472 (55.7) |
| Median BMI, kg/m^2^ [IQR] | 24.8 [20.9-28.6] | 24.81 [21.5-28.5] | 24.82 [21.9-28.9] |
| **Clinical presentation** |  |  |  |
| Fever, n (%) | 454/537 (84.5) | 1798/2119 (84.9) | 383/472 (81.1) |
| Myalgia, n (%) | 118/536 (22.0) | 538/2104 (25.6) | 100/472 (21.2) |
| Cough, n (%) | 408/537 (76.0) | 1652/2118 (78.0) | 373/472 (79) |
| Dyspnea, n (%) | 327/365 (89.6) | 1141/1493 (76.4) | 330/410 (80.5) |
| **Recent influenza vaccination, n (%)** | 319/535 (59.6) | 916/2097 (43.7) | 245/471 (52.0) |
| **Chronic diseases** |  |  |  |
| Chronic pulmonary disease, n (%) | 332/365 (91.0) | 523/1497 (34.9) | 201/410 (49.0) |
| Chronic heart disease, n (%) | 226/535 (42.2) | 871/2118 (41.1) | 212/472 (44.9) |
| Malignancy, n (%) | 87/535(16.3) | 371/2118 (17.5) | 67/472 (14.2) |
| Immunosuppression, n (%) | 41/536 (7.6) | 168/2118 (7.9) | 32/472 (6.8) |
| Chronic kidney disease, n (%) | 71/536 (13.2) | 319/2119 (15.1) | 84/472 (17.8) |
| Mellitus diabetes, n (%) | 118/537 (22.0) | 484/2119 (22.8) | 129/472 (27.3) |
| **Associated treatments** |  |  |  |
| Inhaled corticosteroids, n (%) | 537/537 (100.0) | 0/2121 (0.0) | 111/472 (23.5) |
| Systemic corticosteroids, n (%) | 104/537 (19.4) | 229/2121 (10.8) | 50/472 (10.6) |
| Immunosuppressive drugs, n (%) | 38/536 (7.1) | 204/2119 (9.6) | 39/472 (8.2) |
| Active smoking, n (%) | 79/573 (14.3) | 317/2121 (14.9) | 84/472 (17.8) |
| **Clinical outcomes** |  |  |  |
| In-hospital mortality, n (%) | 17/537 (3.2) | 96/2121 (4.5) | 25/472 (5.3) |
| ICU admission, n (%) | 67/537 (12.5) | 178/2121 (8.4) | 52/472 (11.0) |
| Mechanical ventilation, n (%) | 33/223 (14.8) | 92/821 (11.2) | 36/173 (20.8) |
| Pneumonia, n (%) | 171/535 (32.0) | 638/2115 (30.2) | 110/468 (23.5) |
| Respiratory failure, n (%) | 202/535 (37.8) | 576/2115 (27.2) | 94/468 (20.1) |
| ARDS, n (%) | 48/535 (9.0) | 176/2114 (8.3) | 35/468 (7.4) |
| Heart failure, n (%) | 75/535 (14.0) | 302/2113 (14.3) | 44/468 (9.4) |
| Renal failure, n (%) | 63/535 (11.8) | 297/2115 (14.0) | 42/468 (9.0) |
| Shock, n (%) | 9/535 (1.7) | 86/2114 (4.1) | 11/468 (2.4) |

* missing data on inhaled corticosteroids and/or respiratory virus testing; ICU, intensive care unit; ARDS, acute respiratory distress syndrome; BMI, body mass index; IQR, interquartile range

**Supplementary Table S2.** Risk factors for intensive care unit admission in patients admitted for respiratory virus infections: final logistic regression model

| ICU admission |  | Univariate |  | Multivariate |  |
| --- | --- | --- | --- | --- | --- |
| (n=129/1389) | n/N (%) | OR (95%CI) | *P*-value | aOR (95%CI) | *P*-value |
| Age, median [IQR] | 69 [59-78] | 0.98 (0.97-0.99) | 0.0018 | 0.98 (0.97-0.99) | 0.036 |
| Influenza vaccination ^1^ | 52/126 (41.3) | 0.68 (0.46-1.02) | 0.063 |  |  |
| Chronic respiratory disease | 76/129 (58.9) | 2.25 (1.51-3.37) | < 0.0001 | 1.91 (1.13-3.23) | 0.005 |
| Chronic heart disease | 51/129 (39.5) | 0.81 (0.54-1.21) | 0.31 |  |  |
| Malignancy | 22/129 (17.1) | 0.87 (0.51-1.44) | 0.61 |  |  |
| Chronic kidney disease | 21/129 (16.3) | 1.06 (0.61-1.77) | 0.84 |  |  |
| Inhaled corticosteroids | 40/129 (31) | 2.04 (1.30-3.17) | 0.0017 | 1.33 (0.77-2.27) | 0.30 |
| Systemic corticosteroids | 21/129 (16.3) | 1.30 (0.74-2.20) | 0.35 |  |  |
| Immunosuppressive drugs | 18/129 (14) | 1.34 (0.73-2.37) | 0.32 |  |  |
| Probabilistic antiviral treatment ^2^ 25/129 (19.4) | | 8.51 (4.22-18.08) | < 0.0001 | 7.91 (3.7-17.56) | < 0.001 |
| Influenza | 67/129 (51.9) | 0.71 (0.48-1.05) | 0.087 |  |  |
| Respiratory syncytial virus | 24/129 (18.6) | 1.65 (0.96-2.77) | 0.064 |  |  |

^1^ Last 6 months; ICU, intensive care unit; IQR, interquartile range

^2^ oseltamivir started before influenza was documented

**Supplementary Table S3.** Risk factors for adenovirus, respiratory syncytial virus, and influenza among patients hospitalized for respiratory virus infections, as identified in the final logistic regression model

|  | aOR (95%CI) | *P*-value |
| --- | --- | --- |
| **Adenovirus** |  |  |
| Age > 65 years | 0.84 (0.44 - 1.65) | 0.60 |
| Male | 2.04 (1.04 - 4.22) | 0.044 |
| Inhaled corticosteroids | 2.36 (1.18 - 4.58) | 0.012 |
|  |  |  |
| **Respiratory syncytial virus** |  |  |
| Age > 65 years | 1.38 (0.94 - 2.08) | 0.11 |
| Male | 0.87 (0.60 - 1.26) | 0.47 |
| Malignancy | 1.78 (1.14 - 2.73) | 0.01 |
| Inhaled corticosteroids | 2.05 (1.36 - 3.04) | 0.0004 |
|  |  |  |
| **Influenza** |  |  |
| Age > 65 years | 0.73 (0.54 - 0.98) | 0.04 |
| Male | 1.17 (0.90 - 1.54) | 0.23 |
| Chronic respiratory disease | 0.67 (0.50 - 0.91) | 0.0009 |
| Malignancy | 0.63 (0.45 - 0.89) | 0.007 |
| Seasonal influenza vaccination | 0.73 (0.55 - 0.96) | 0.03 |
| Inhaled corticosteroids | 0.87 (0.6 - 1.25) | 0.45 |

The outcome analyzed were adenovirus, respiratory syncytial virus, and influenza in a backward logistic regression.

Models were adjusted for age, and sex. aOR: adjusted odds ratio; 95%CI: 95% confidence interval
